# Finding Common Ground: Environmental Ethics, Social Justice, and a Sustainable Path for Nature-Based Health Promotion

**DOI:** 10.3390/healthcare4030061

**Published:** 2016-08-25

**Authors:** Viniece Jennings, Jessica Yun, Lincoln Larson

**Affiliations:** 1 Southern Research Station, Integrating Human and Natural Systems, USDA Forest Service, 320 Green Street, Athens, GA 30602, USA; 2 Department of Environmental Planning, Graduate School of Environmental Studies, Seoul National University, Building 82, 1 Gwanak-ro, Gwanak-gu, Seoul 08826, Korea; jessjyun@snu.ac.kr; 3 Department of Parks, Recreation, and Tourism Management, Clemson University, 298 Lehotsky Hall, Clemson, SC 29634, USA; lrl@clemson.edu

**Keywords:** nature, public health, green space

## Abstract

Decades of research have documented continuous tension between anthropocentric needs and the environment’s capacity to accommodate those needs and support basic human welfare. The way in which society perceives, manages, and ultimately utilizes natural resources can be influenced by underlying environmental ethics, or the moral relationship that humans share with the natural world. This discourse often centers on the complex interplay between the tangible and intangible benefits associated with nonhuman nature (e.g., green space), both of which are relevant to public health. When ecosystem degradation is coupled with socio-demographic transitions, additional concerns related to distributional equity and justice can arise. In this commentary, we explore how environmental ethics can inform the connection between the ecosystem services from green space and socially just strategies of health promotion.

## 1. Introduction

A transformative perspective on public health requires insight from the environmental field [1]. From Hardin’s seminal essay on the tragedy of the commons [2] to the contemporary discussion on public goods [3], society is reminded that natural resources are limited and represent a critical component of the system that supports the human condition. Likewise, one’s perception, management, and use of natural resources, all of which are inherently cultural, can be influenced by environmental ethics, a branch of philosophy that explores the moral relations between humans and nonhuman nature [4,5]. While environmental ethics has many theoretical components, the field has practical applications as well. For example, environmental ethics includes axiology, the study of values and valuation, which can relate to other duties and obligations within the human-nature relationship [6]. Just as social ethics can guide how humans behave towards each other, environmental ethics can guide how humans relate to and manage the natural resources that surround them.

For many scholars, especially economists and environmental scientists, anthropocentric conversations about nature’s value typically revolve around the benefits it provides to people, which are often described as ecosystem services. Through this lens, the value of nature is often assessed through factors and processes that generate tangible outputs affecting human health and wellbeing [7,8,9]. Such a pragmatic approach is undoubtedly important, but environmental ethics suggests the value of nature and ecosystem services transcends these purely quantitative metrics [6]. Although many conservation biologists laud the intrinsic value of nature in their protection efforts, this biocentric/ecocentric message [10] rarely generates a defensible stance that resonates with other audiences [11]. Society's adoption of ethical viewpoints that explore the overlap between environmental conservation and public health can support sustainable interactions between humans and nature in a way that encourages healthy environments for all. This article examines the role of environmental ethics in health promotion by exploring the complex concept of ecosystem services and considering the health implications of non-human nature (e.g., green spaces) through an environmental justice perspective.

## 2. Ecosystem Services and Public Health Benefits

Nature provides an array of ecosystem services (e.g., water purification, timber, sources of food, outdoor recreation) that highlight inextricable links between humans and the ecosystems that support their health [12]. The ecosystem services framework can also encourage a systems thinking approach which recognizes the different components within a given system. For instance, the provision of ecosystem services is mediated through biophysical factors, ecological structures, and processes which result in outputs from nature that can be beneficial to humans. Changes to ecological structure and functions have a range of human health implications that researchers should continue to explore [13]. As an illustration, green spaces provide various ecosystem services (e.g., aesthetic surroundings, shade, etc.) that can alleviate health challenges such as obesity, cardiovascular disease, mental health concerns, and heat-related illness [14]. However, these ecosystem services often exhibit spatial and temporal variation due to factors such as overall environmental quality, ecological change, and socio-demographic transitions. Therefore, a vision of sustainable health care through environmental ethics can promote a holistic approach to health that considers the stability of human populations and the natural environment [15]. Understanding the nuances of this relationship can be supported by an ecosystem services framework which describes the multitude of ways that the natural environment supports human health and well-being.

While many scholars acknowledge the fundamental importance of access to quality health care, ecosystem services from green spaces also enhance characteristics of place in ways that influence determinants of health and ultimately encourage health promotion [9]. In their recent review article, Mantler and Logan [16] express how the benefits from green space can have clinical implications for mental health care. Other frameworks articulate how the natural environment [17] and the ecosystems therein [18] are interconnected with other domains of health and well-being. Growing evidence suggests that neglect for nonhuman nature might also negatively impact multiple aspects of human development [19,20]. For example, research has shown that air quality may be linked to cognitive development [21], autism [22], and an array of other neuropsychological disorders [23]. Other research demonstrates links between green space, physical activity, and cardiovascular health [24,25]. Meanwhile, studies also elaborate on the nature–health relationship in the context of “doses” of nature [26], health disparities across sociodemographic groups [14], and nature-based health interventions [27]. On the surface, existing literature highlights clear benefits of nature to public health. However, due to the scarcity of longitudinal studies on this topic and the challenges of defining recommended “doses” of nature, additional research is needed to understand the benefits of nature and their potential value as a preventative health promotion tool [16]. Since all green spaces possess different attributes [14,27], tradeoffs between beneficial services and disservices should also be considered. For example, green spaces that promote healthy, active lifestyles (via park-based physical activity and stress reduction) may also exacerbate pollen emissions, which negatively affect certain segments of the population with allergies. Additionally, many scholars acknowledge that complexity within the ecosystem services framework can lead to different interpretations depending on factors such as geographic scale and management goals [6]. Because of this persistent uncertainty regarding quantification of impacts, equity of distribution, and potentially contentious analysis of costs and benefits, environmental ethics becomes an important piece of the puzzle.

## 3. Ecosystems’ Public Health Benefits and the Role of Environmental Ethics

One of the most fundamental issues in environmental ethics is the debate regarding the valuation of nonhuman nature [6,28]. While some scholars claim that nonhuman nature has an intrinsic (or inherent) moral value independent of the existence of humans, others advance nature’s non-intrinsic values to humans in a variety of ways [6]. Instrumental value, which has historically dominated economic discourse, views nature as a replaceable and compensable means to achieve something else [6]. With their emphasis on tangible outputs (e.g., food, climate regulation, pollution control) and commodification of nature [29], ecosystem service frameworks are firmly rooted in these instrumental valuation perspectives. However, to obtain a more comprehensive understanding of the relationship between human and nonhuman nature, a broader range of values could be considered [6]. For example, fundamental value refers to acknowledging the worth of nature as essential to the condition of life and not merely a means to increase utility [6]. Meanwhile, eudaimonistic value refers to recognizing nature for its ability to improve humans' general quality of life, usually in terms of leisure and aesthetic experiences [6]. Many studies, for example, document how green space can be positively associated with happiness [30] and other measures of subjective well-being [8]. Integrating such perspectives on the valuation of nonhuman nature can improve how environmental ethics are merged with frameworks in ecosystem services and health promotion.

How might the concepts of ecosystem services and environmental ethics be integrated? In a recent study, Bull et al. [31] conducted a survey of environmental specialists to perform a SWOT analysis of strengths, weaknesses, opportunities, and threats associated with the ecosystem services framework. This SWOT analysis revealed that the concept’s ambiguous language and disregard for nature’s intrinsic value are some of its potential weaknesses [31]. Results from their survey also noted that a perspective in environmental ethics can threaten the ecosystem services framework since the latter elevates the instrumental values based on human needs over the intrinsic value of nature [31]. Another study [6] expressed concerns that monetizing ecosystem services can be misleading since it minimizes a number of environmental benefits (e.g., biodiversity) that cannot be properly captured in an exchange unit. While efforts to connect the value of nature to utilitarian human concerns may accentuate their relevance to society, this perspective is somewhat limiting. For example, more people in developed countries embrace a balanced perspective that recognizes the intrinsic value of nature and wildlife as a contributor to ecological and human health [32].

In order to fully realize nature’s vital role in public health, Horton et al. [1] encourage us to transform our perspective toward natural resources and how they might contribute to human health and well-being. For example, Jameton and Pierce [15] note that few scholars acknowledge that provision of health care, while heavily dependent on a healthy environment, is also a source of environmental degradation (i.e., waste, use of natural resources). Some hospitals recognize this negative feedback loop and have become involved in sustainability initiatives that integrate core values such as managing resources and reducing waste as an important aspect of healthcare [33]. Consequently, values embodied by environmental ethics may precipitate refinement of visions for sustainable health care that transcend traditional boundaries. For example, the way that ecosystem services are presented in public health arenas can greatly influence how nature’s benefits are translated and valued across disciplines and cultures. For example, Jennings et al. [9] connected the ecosystem services from green space to benefits that support different social determinants of health. Along similar lines, Robinson and Elliott [34], describe how the environment’s “aesthetic integrity” can promote community attachment and well-being in a way that effectively conveys the value of natural resources. On the other hand, environmental conservation should be grounded in an ethical base that acknowledges both the intrinsic and instrumental value of nature [11]. Understanding the value of nature from different perspectives can enhance our understanding of the complex socio-ecological systems that influence human health and well-being.

## 4. Environmental Justice as an Expression of Environmental Ethics

The environmental justice movement, which stemmed from concerns that environmental burdens (e.g., landfills, toxic emitting facilities, etc.) were disproportionately located in minority communities and economically impoverished neighborhoods [35], also seeks to reduce inequitable distribution of environmental benefits to humans [36]. As issues in justice prompt essential ethical concerns [6], environmental ethics becomes intertwined with the field of environmental justice. However, the underlying role of ethics in environmental justice may not be obvious or explicitly shown in the literature. Despite limited dialogue between these areas through the years, an ethical perspective on environmental justice can be beneficial to both fields [37]. Some scholars view environmental justice as an interdisciplinary synergy that links environmental ethics with other fields such as public administration, political theory, and human ecology [38]. Others see environmental justice as “the social justice expression” of environmental ethics [39]. In other words, environmental ethics can fortify the moral fabric of the environmental justice movement. Meanwhile, environmental justice can demonstrate the social facets of environmental ethics in an applied context [37]. For instance, restorative justice represents an ethical framework in which the injustices faced by marginalized groups are acknowledged and honored by others [40]. McDermott et al. 2013 [41] also advocate for effective means to achieve procedural and contextual justice to promote equitable management of ecosystem services. These forms of justice entail fairness in the political process that distributes ecosystem services and consideration of existing barriers that can limit the capacity to receive benefits, respectively [41].

A critical component underlying both environmental justice and health equity is the broader social ethics concept of distributive justice. Rawls [42] advocated for societies to maximize the well-being of the least well-off individual. This concept can also be applied in the pursuit of equitable health promotion. Poor health of individuals in certain neighborhoods would further disadvantage them socially and economically, making health equity an issue of social justice [43]. Thus, to holistically achieve distributive justice, protection of the health of the most socio-economically disadvantaged must be prioritized. However, Rawls's theory of justice has also received a fair share of criticism from both libertarians and communitarians alike [44]. For example, libertarians have argued that the well-off have no moral obligation to help the least well-off [45]. Yet equitable provision of healthcare should be a central tenet of health promotion systems. Considering the important links between nature and health outlined above, equitable provision of green space is just as important [46].

Access to green spaces was not initially considered in traditional environmental justice research, but this paradigm has expanded to consider equitable access to parks and other health-promoting natural amenities [47,48]. For example, recent studies document how socio-economic factors such as race/ethnicity [49,50] and income [50] are associated with the distribution of tree cover. Limited recreation opportunities for disadvantaged populations also have a number of social justice implications and, according to some scholars, represents a moral imperative for inquiry in leisure studies [51]. Since social justice plays a fundamental role in sustainable development and public health [52], it provides an important lens through which environmental issues can be viewed. In their commentary on social justice, ethics, and public health, Goston and Powers [52] explained that justice “captures the twin moral impulses that animate public health: to advance human well-being by improving health and to do so by focusing on the needs of the most disadvantaged.” Moreover, a social justice approach to public health can advance the field by reducing health disparities and other negative determinants of health within the most vulnerable populations [52]. For example, the availability and quality of ecosystem services derived from nonhuman nature can influence environmental health disparities across sociodemographic groups [14]. While the perspectives of communities that can most benefit from ecosystem services are rarely heard, the values that influence the management of ecosystem services also intersect with justice concerns [6]. Thus, it is also important for environmental justice to merge with different paradigms such as environmental ethics [51] and ecosystem services to effectively address health concerns in underserved communities.

## 5. Balancing Environmental Ethics, Environmental Justice and Health

**Figure 1:**
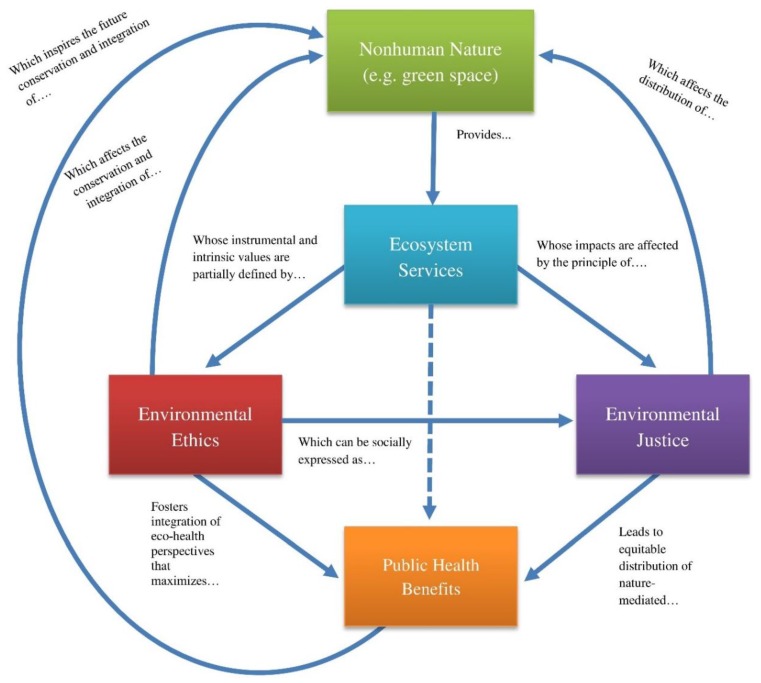
Conceptual map illustrating the connections among nonhuman nature, ecosystem services, environmental ethics, environmental justice, and public health.

Figure 1 depicts conceptual links between nonhuman nature (and the ecosystem services it provides), environmental ethics, environmental justice, and public health. While the flowchart has no essential starting point, one might begin by examining the critical capacity of nonhuman nature to provide ecosystem services. These ecosystem services generate instrumental and intrinsic values that ultimately produce public health benefits, which in turn enhance the perceived value of nature. However, the extent of these benefits and the way in which they are expressed across different populations is mediated by two important intersecting concepts: environmental ethics and environmental justice. Environmental ethics influence decisions that affect the conservation of ecosystem services that are valued for a variety of reasons (e.g., economically, sentimentally, and aesthetically). Environmental ethics also influences the social expression of environmental justice, which affects the provision of ecosystem services and the distribution of corresponding health amenities (e.g., green space) across diverse communities. The dotted path between ecosystem services and public health benefits represents a conventional, unidimensional path between nature and health that fails to adequately account for key factors such as environmental conservation and social justice. Such oversight can be problematic, and may exacerbate health disparities. Environmental ethics and environmental justice therefore represent critical elements in proactive healthcare frameworks that integrate ecological systems, ultimately producing a self-sustaining path to health promotion.

Simultaneous integration of these concepts can be challenging, however. In their article on ethical responsibilities related to environmental health, Jameton and Pierce [15] outlined three challenges that can lead to ethical tensions between health care and environmental issues. These tensions include balancing individual needs with the greater whole, achieving sustainability through social justice, and maintaining quality health with sustainability pursuits [15]. The first challenge refers to a healthcare providers’ commitment to helping patients while minimizing damage to the natural world. For example, some entities monitor energy consumption [33] or use ecological footprints to quantify the use and potential degradation of natural resources to consumers [15]. Remedies to this challenge recognize that social justice and a holistic vision of sustainability are complementary goals that are essential to maintaining population health [15]. The second challenge considers the combination of poverty, limited resources, and strained ecosystems and examines if and how visions of justice and sustainability can be simultaneously achieved [15]. Some scholars argue that neither environmental sustainability nor social justice can override one another [53] and both perspectives are critical to support sustainability in a socially just way [54]. There is also a need for more scholars in environmental ethics to explore issues related to environmental justice [37]. Similarly, the third challenge acknowledges the task of stabilizing global health with sustainability pursuits. However, this dilemma also realizes that destructive economic development at the expense of environmental quality creates a feedback loop that contradicts the ideals of sustainable development [15]. At the same time, continuous economic expansion without regard to the environment and public health can inadvertently promote inequities and reduce citizens' quality of life. Therefore, professionals in urban planning, ecology, outdoor recreation, and public health could focus on urban green space strategies that are “just green enough”, concurrently protecting both social and ecological sustainability as a means of promoting public health [55].

Other research has examined the convergence of ethics in ecosystem and health management. Humphreys et al. [40] articulate how integrating an ethical framework of restorative justice within ecosystem management can recognize social context and value diverse populations and their perspectives. This approach can also re-position ecosystem management within the scope of social concerns, extending beyond the sole emphasis on the natural sciences that has historically dominated ecological discourse [40]. For instance, expanding access to and use of green space may address the various tensions between health care and environment outlined by Jameton and Pierce [15]. However, since ecosystem services were conceived in the context of the environmental science and ecological economics, the concept was not necessarily designed to translate to clinical interventions in the medical arena [56]. Nonetheless, strategically integrating ecosystem services will be critical for environmental and health professionals hoping to acknowledge and understand potential areas of common ground. Some doctors are already acknowledging the value of nature as a health promotion tool and starting to prescribe park-based recreation as an alternative form of preventative medical care [56,57]. In order to further promote sustainable healthcare practices, it is important to engage healthcare professionals in the dialogue surrounding environmental ethics and the provision of health services in an environmentally responsible way [15]. Additionally, establishing or revitalizing parks in areas lacking green space could promote social justice and sustainability by providing nature-based physical and mental health benefits to those communities while also supporting local biodiversity and ecological networks [58].

## 6. Conclusions

Environmental ethics and environmental justice are the transformative lenses through which ecosystem services are leveraged to yield sustainable health outcomes for individuals from all backgrounds. Cultivation of an environmental ethic may support the valuation and ultimate conservation of important ecosystem services from both anthropocentric and biocentric/ecocentric perspectives. Similarly, strengthening the ethical foundation of justice concerns can support effective policy implementation and concerns related to environmental injustice [37], thereby impacting the provision of nature-related health amenities and disservices. It is therefore vital to incorporate multiple levels of environmental awareness and stewardship into health care education and administration [15]. The values and moral responsibilities that are expressed in environmental ethics can influence our perception of nonhuman nature, environmental justice, and their relationship to public health. Others recommend further integration of environmental concerns in medical education to develop an environmental consciousness in fields such as preventive medicine [15,56,59]. Since many factors that affect health are regulated outside the realms of health agencies [60], it is critical to truly approach health from an interdisciplinary perspective. The ideals of fairness, equality, and justice, particularly in an environmental context, should be guiding principles to steer research in a variety of fields [51] that have public health implications [15]. A symbiotic conversation on social justice, ecosystem services, and health can evolve as these linkages are made, thereby generating insights that support human populations, present and future.

## Abbreviations

The following abbreviation is used in this manuscript: SWOT: Strengths, Weaknesses, Opportunities and Threats.
